# Kernel current source density method

**DOI:** 10.1186/1471-2202-12-S1-P375

**Published:** 2011-07-18

**Authors:** Jan Potworowski, Wit Jakuczun, Szymon Łęski, Daniel K Wójcik

**Affiliations:** 1 Laboratory of Neuroinformatics, Nencki Institute of Experimental Biology, Warsaw, 02-093, Poland; 2 WLOG Solutions, Warsaw, 02-389, Poland

## 

Local field potentials (LFP), the low-frequency part of extracellular electric potential, reflect dendritic processing of synaptic inputs to neuronal populations. They are an invaluable tool in the studies of neural activity both in vivo and in vitro. With recent fast development of multielectrode technology one can easily record potentials in different geometries, including 3D setups, from multiple sites simultaneously. Due to the long-range nature of electric field each electrode may reflect activity of sources located millimeters away which complicates analysis of LFP. Whenever possible it is convenient to estimate the sources of measured potential, called current source density (CSD), which is the volume density of net transmembrane currents. CSD directly reflects the local neural activity and current source density analysis is often used to analyze LFP.

In homogeneous and isotropic tissue CSD is given by the Laplacian of the potentials, so discrete differentiation is the simplest estimate for a set of potentials on a regular grid. Recently continuous methods for CSD estimation have been developed, called the inverse CSD (iCSD). These methods assume a specific parametric form of CSD generating potentials and calculate the LFP in a forward-modeling scheme to obtain the values of CSD parameters. The iCSD framework assumes CSD distributions parameterized with as many parameters as there are measurements.

**Figure 1:**
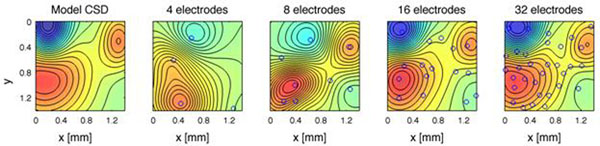
CSD reconstructions from randomly placed electrodes. Model data and reconstructions from 4, 8, 16, and 32 randomly placed electrodes

Here we present a nonparametric method for CSD estimation based on kernel techniques. Kernel Current Source Density method lets the user specify the family of allowed CSD distributions through a basis of dimensionality much larger than the number of measurements. Prior knowledge of the anatomy or physiology of the probed structure, such as laminarity, can be incorporated in the method. kCSD can be applied to recordings from electrodes distributed arbitrarily on one-, two-, and three-dimensional sets so one can consider experimental setups optimally adapted to a research problem of interest (Figure 1). We show that kCSD is a general non-parametric framework for CSD estimation including all the previous variants of iCSD methods as special cases.
